# Sonic Hedgehog Signaling Pathway Mediates Proliferation and Migration of Fibroblast-Like Synoviocytes in Rheumatoid Arthritis *via* MAPK/ERK Signaling Pathway

**DOI:** 10.3389/fimmu.2018.02847

**Published:** 2018-12-05

**Authors:** Fang Liu, Xiao Xue Feng, Shang Ling Zhu, Hong Yu Huang, Ying Di Chen, Yun Feng Pan, Rayford R. June, Song Guo Zheng, Jian Lin Huang

**Affiliations:** ^1^Division of Rheumatology, Department of Internal Medicine, The Sixth Affiliated Hospital, Sun Yat-sen University, Guangzhou, China; ^2^Division of Rheumatology, Department of Internal Medicine, The Third Affiliated Hospital Sun Yat-sen University, Guangzhou, China; ^3^Faculty of Arts and Science, University of Toronto, Toronto, ON, Canada; ^4^Division of Rheumatology, Milton S. Hershey Medical College at Penn State University, Hershey, PA, United States

**Keywords:** arthritis, rheumatoid, Sonic Hedgehog, mitogen-activated protein kinases, cell proliferation, cell migration, MAPK/ERK, signal transduction

## Abstract

Fibroblast-like synoviocytes (FLSs) are the major effector cells that lead to rheumatoid arthritis (RA) synovitis and joint destruction. Our previous studies showed that Sonic Hedgehog (SHH) signaling pathway is involved in aberrant activation of RA-FLSs and inhibition of SHH pathway decreases proliferation and migration of RA-FLSs. The objective of this study was to investigate if the SHH pathway mediates proliferation and migration of RA-FLSs *via* the mitogen-activated protein kinases/extracellular signal-regulated kinases (MAPK/ERK) signaling pathway. SHH signaling was studied by using SHH agonist (Purmorphamine) and antagonist (Cyclopamine) targeting the Smoothened (SMO) in FLSs. U0126-EtOH was used to inhibit the MAPK/ERK signaling pathway. The phosphorylation of ERK 1/2 (p-ERKl/2) was examined by western blot. Cell viability was detected using cell proliferation and cytotoxicity kit-8 (CCK8), and cell cycle distribution and proliferating cells were evaluated by the flow cytometry. Cell migration was examined by Transwell assay. Results showed that, compared with the control group, Purmorphamine increased the levels of p-ERK1/2 in concentration-and time-dependent manners (*P* < 0.01). Co-treated with Purmorphamine and U0126-EtOH or Cyclopamine both decreased the levels of p-ERK1/2 (*P* < 0.05). RA-FLSs treated with Purmorphamine resulted in alteration of cell cycle distribution, increasing of proliferating cells, cell viability, and migration cells compared to controls (*P* < 0.01). However, the above phenomenon can be abolished by U0126-EtOH (*P* < 0.05). The findings suggest that SHH signaling pathway mediates proliferation and migration of RA-FLSs *via* MAPK/ERK pathway and may contribute to progression of RA. Targeting SHH signaling may have a therapeutic potential in patients with RA.

## Introduction

Rheumatoid arthritis (RA) is a chronic inflammatory joint disease, which can cause cartilage and bone damage as well as disability (1). The main pathological feature of established RA is synovial hyperplasia where activated fibroblast-like synoviocytes (FLSs), together with the accumulated activated T cells, activated B cells and monocytes create tissue proliferation causing articular joint destruction (2). FLSs display surprisingly pathogenic behavior (3), including increasing in number and becoming a prominent component of the destructive pannus (4). Furthermore, FLSs in RA acquire an aggressive phenotype and can potentially migrate from joint to joint to propagate disease (5). Proliferation and migration are two fundamental properties of RA-FLSs that contribute to the pathological process of RA. Many signaling pathways are thought to be involved in the proliferation and migration of RA-FLSs, including the Janus kinase/signal transducer and activator of transcription (JAK/STAT) pathway (6), nuclear factor-kappaB (NF-κB) pathway (7), mitogen-activated protein kinases (MAPKs) pathway (8).

Hedgehog (HH), including sonic hedgehog (SHH), Indian hedgehog (IHH), and desert hedgehog (DHH), appear to bind to a transmembrane receptor protein, known as Patched (PTCH), which in the absence of HH suppresses smoothened (SMO) activity (9). Binding of HH to PTCH activates SMO, and induces a complex series of intracellular reactions *via* activation of GLI transcription factors (GLI1-3) and their target genes (10). The SHH signaling pathway is a conserved signaling system essential for embryonic development and tissue differentiation. Target genes in the SHH signaling pathway are related to cell proliferation, survival, cell cycle, stem cell formation, cell invasion and many other processes (11).

Our previous studies have shown that SHH signaling pathways are abnormally active in RA synovial tissue and RA-FLSs (12). Moreover, SHH signaling pathway is correlated to the proliferation and migration of RA-FLSs in a manner of depending on SMO (13, 14). However, the specific mechanism by which SHH signaling pathway is involved in proliferation and migration of RA-FLSs remains unknown.

Many studies show an association between SHH signaling pathway and MAPK/ERK signaling cascade. For example, autocrine SHH regulates the proliferation of gastric cancer cells *via* MAPK/ERK signaling pathway (15). Furthermore, the SHH pathway mediates invasion and metastasis hepatocellular carcinoma *via* the ERK pathway (16). These data suggest that the signal transduction through SHH signaling pathway is closely associated with the activation of intracellular MAPK/ERK cascade, which is the downstream effector of many important signaling pathways, such as the epidermal growth factor receptor (EGFR) signaling pathway (17). However, it is unclear the mechanisms in which the SHH signaling pathway regulates proliferation and migration of RA-FLSs and if the MAPK/ERK signaling pathway is involved.

In the present studies, we investigated the mechanisms of the SHH signaling pathway in proliferation and migration of RA-FLSs, specifically focusing on the effects of SHH signaling on the activation of MAPK/ERK signaling pathway.

## Materials and Methods

### Patients

Han Chinese patients with active RA, including two males and five females (mean age 54.42 ± 6.45 years) were recruited from the Third Affiliated Hospital of Sun Yat-sen University in Guangzhou, China, from April 2015 to February 2016. Synovial tissues were obtained by the synovectomy. RA patients were classified according to the 1987 American College of Rheumatology revised classification criteria and exhibited moderate to severe disease activity (Disease Activity Score of 28 joint counts > 3.2) (18). This study was approved by the Medical Ethics Committee of the Third Affiliated Hospital of Sun Yat-sen University. All patients gave written informed consent.

### Cell Culture and Cell Characterization

FLSs were isolated and cultured from RA synovium. Tissue biopsies were finely minced into pieces and transferred to a tissue culture flask in Dulbecco's modified Eagle's medium (DMEM) (Gibco Laboratories, Invitrogen, USA) supplemented with 10% fetal bovine serum (FBS) (Gibco Laboratories). Within 14 days, FLSs migrated out from the tissue explant and were grown to approximately 95% confluency. FLSs were subsequently trypsinized, collected, re-suspended, and planted for proliferation. FLSs from passages 3–5 were used for the following experiments.

The morphological characters of FLSs were observed under the light microscope. The expression level of surface markers on FLSs were detected for characterization using flow cytometry. FLSs from passages 3 were trypsinized, centrifuged, and stained with commercial monoclonal antibodies CD68FITC, CD14FITC, CD90FITC, CD55PE (Biolegend, USA) for 20 min. And isotype-matched control antibodies were used as methodologic controls. Stained cells were subsequently analyzed using a FACSCalibur Flow Cytometer (Becton Dickinson, Franklin Lakes, NJ, USA). For each analysis, 10,000 events were evaluated with the software FlowJo 7.6 (Becton Dickinson, Franklin Lakes, NJ, USA).

### RNA Isolation and Real-Time PCR Analysis

To measure the effects of Cyclopamine and Purmorphamine on SHH signaling, GLI1 and SMO expression was determined by real-time PCR analysis. FLSs were plated at a density of 5 × 10^4^ mL-1 in 6-well plates for 24 h and then treated with Cyclopamine (10 μM) or Purmorphamine (1 μM) for 48 h. Total RNA was isolated using Trizol reagent (Invitrogen Life Technologies, Santa Clara, CA, USA) and cDNAs were synthesized using the Prime Script RT Reagent kit (Takara Biotechnology, Dalian, China) according to the manufacturer's instructions. All experiments were examined in triplicate and positive (sample from liver cancer cells containing SMO or GLI1 nucleotide sequence) and negative (sterile deionized water containing no template) controls were included. Quantification of expressions of human GLI1, SMO, and GAPDH mRNAs was determined using SYBR Premix Ex Taq™ kit (Takara Biotechnology) on an ABI-7500 Thermal Cycler (Applied Biosystems Inc., Foster City, CA, USA) according to the manufacturer's instructions. Relative levels were quantified by the comparative delta C method. Primers for amplification were as follows (forward,reverse): SMO:(5′-CCTGCTCACCTGGTCACTC−3′,5′-CACGGTATCGGTAGTTCTTGTAG-3′),GLI1:(5′-AGGGAGTGCAGCCAATACAG-3′,5′-CCGGAGTTGATGTAGCTGGT-3′),GAPDH:(5′-GCACCGTCAAGGCTGAGAAC−3′,5′-TGGTGAAGACGCCAGTGGA-3′).

### Western Blot Analysis

Briefly, total protein was extracted using a lysis buffer (Cell Signaling Technology, Beverly, MA, USA) and phosphatase inhibitors (Roche, Basel, Switzerland). Protein lysates (35 mg protein) were loaded and separated using 8% sodium dodecylsulfate-polyacrylamide gel electrophoresis, and then blotted onto a polyvinylidene fluoride (PVDF) membrane. Membranes were blocked at room temperature for 2 h and incubated overnight at 4°C with primary antibodies. Primary antibodies included rabbit anti-SMO (1:500, Abcam, Cambridge, UK) and the phosphorylation extracellular signal-regulated kinases (p-ERK1/2) (1:2,000, Cell Signaling Technology, Beverly, MA, USA). Subsequently membranes were incubated for 1 h at room temperature with secondary antibodies conjugated with horseradish peroxidase. The expression of GAPDH or ERK1/2 was used as an internal standard. The immobilized proteins were measured by the enhanced chemiluminescent (ECL) detection system. The band density was quantified by ImageJ2x software.

### Cell Viability Assay

FLSs were seeded at a density of 2.5 × 10^4^ mL^−1^ in 96-well plates. Twenty-four hours later, cells were treated with SHH antagonist (Cyclopamine, Selleckcem, Houston, TX, USA) (10 μM) or SMO agonist (Purmorphamine, Sigma-Aldrich, St. Louis, MO, USA) (1 μM), or co-treated with U0126-EtOH (Selleckcem, USA) and Purmorphamine (1 μM) cultured for another 48 h. Cell proliferation rates were subsequently assessed using the cell counting kit-8 (CCK-8) (Dojindo, Tokyo, Japan) according to the manufacturer's instructions. Cyclopamine and Purmorphamine were dissolved at 10 mM in dimethyl sulfoxide (DMSO) and the solution was diluted to the final concentration in DMEM supplemented with 10% FBS. Cells in the control group were treated with vehicle (DMSO in DMEM supplemented with 10% FBS).

### Cell Cycle Analysis

For cell cycle phase analysis, FLSs were plated at a density of 5 × 10^4^ mL^−1^ in 6-well plates for 24 h. FLSs were serum starved for 24 h before incubation with Cyclopamine (10 μM) or Purmorphamine (1 μM), or co-treated with U0126-EtOH (10 μM) Purmorphamine (1 μM). After treated for 48 h, FLSs were harvested and fixed in 70% cold ethanol overnight at −20°C. Fixed cells were subsequently washed in phosphate-buffered saline (pH 7.4) and incubated with RNase A (100 μg mL^−1^) (Invitrogen, Carlsbad, CA, USA) for 30 min at 37°C. For staining of nuclei, cells were incubated with propidium iodide (50 μg mL^−1^) (Sigma-Aldrich, St. Louis, MO, USA) in the dark for 30 min at 4°C. Stained cells were subsequently analyzed using a FACSCalibur Flow Cytometer. For each analysis, 10,000 events were evaluated with the software ModFit LT (Verity Software House, Topsham, ME, USA).

### Cell Proliferation Assay

5-Ethynyl-29-deoxyuridine (EdU) is a molecule that is readily incorporated into cellular DNA during its replication and can be detected by flow cytometry. In this study, the EdU assay was performed to evaluate the cell proliferation rate. FLSs were plated at a density of 5 × 10^4^ mL^−1^ in 6-well plates. Twenty-four hours later, cells were treated with Cyclopamine (10 μM), Purmorphamine (1 μM), or co-treated with U0126-EtOH and Purmorphamine cultured for another 48 h, respectively. The proliferating cells were subsequently assessed using the Cell-Light™EdU Apollo®643 *in vitro* Flow Cytometry Kit (Ribobio, Guangzhou, China) according to the manufacturer's instructions. The FACSCalibur Flow Cytometer was used to detect the ratio of proliferating cells. For each analysis, 20,000 events were evaluated with the software FlowJo 7.6.

### Cell Migration

Migration ability of FLSs was measured in a Transwell cell culture chamber apparatus with 8 μm pore membrane (Costar, New York, NY, USA). Briefly, FLSs were seed at a density of 5 × 10^4^ mL^−1^ in six-well plates, and were treated with Cyclopamine (10 μM), Purmorphamine (1 μM), or co-treated with U0126-EtOH and Purmorphamine cultured for another 48 h, respectively. Then, FLSs were trypsinized, collected, and re-suspended with serum-free medium. The cell suspension (5 × 10^3^ mL^−1^) was loaded into the upper chamber of the Transwell insert. Medium containing 10% FBS (600 μL) was added to the lower compartment as a chemoattractant. After 8 h of incubation, the filters were removed and cells remaining on the upper surface of the membrane were removed with a cotton swab. The cells adhering beneath the membrane were fixed in 4% paraformaldehyde and stained with crystal violet for 30 min. Migration ability of FLSs was quantified by cell counts of five random fields at 100 magnifications in each membrane.

### Statistical Analysis

SPSS statistical software, version 20.0 (Chicago, IL, USA), was used for all statistical analyses. The experimental data were presented as means ± standard deviation (S.D.) or median values and interquartile ranges (IQR) based on ≥3 replicates. Statistical differences among groups were tested by one-way analysis of variance (ANOVA) or the Kruskal–Wallis test. The Dunnett's *t*-test was used for multiple comparisons. Differences were considered statistically significant when *P* < 0.05.

## Results

### Morphological Characters and Surface Markers Expression of FLSs

**Figure 1:**
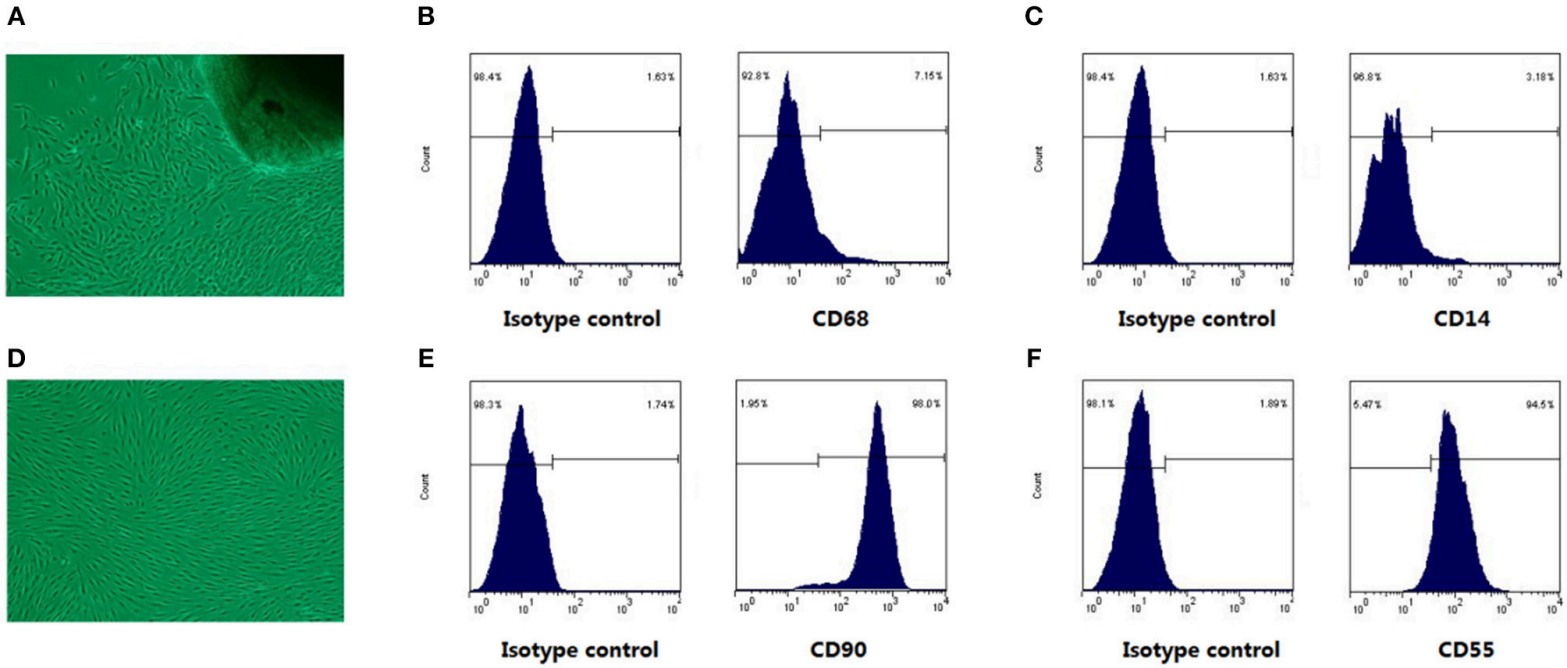
The cell morphological characters and surface markers expression. Under the light microscope, RA-FLSs crawled out from synovial tissues **(A)**, and RA-FLSs at passages 3 cells were spindle-shaped and braided growth **(D)**. In **(B,C,E,F)**, The left was isotype control map; the right was surface mark map. Cells were identified by CD68 **(B)** and CD14 **(C)** negative staining, coupled with positive staining for CD90 **(E)**, CD55 **(F)** staining.

As shown in Figure 1A, the cells crawled out from the edge of the adherent tissue after attachment culture for 3~5 days. By the passage 3, fibroblast-like synoviocytes from RA patients (RA-FLSs) were spindle-shaped and whirlpool-like growth under light microscope (Figure 1D). FLSs are characterized by the high level expression of UDP-glucose 6-dehydrogenase (UDPGDH), VCAM-1, CD55, and CD90 (5), and negative staining for macrophage markers such as CD14 or CD68. The expression level of surface markers (CD90, CD55, CD14, CD68) on FLSs were detected for characterization. The flow cytometry analysis showed that the expression rate of CD90 (Figure 1E) and CD55 (Figure 1F) of FLSs at passage 3 were up to 90%, and the expression rate of CD68 (Figure 1B) and CD14 (Figure 1C) were under 10%.

### Effects of the Purmorphamine and Cyclopamine on SHH Signaling of RA-FLSs

**Figure 2:**
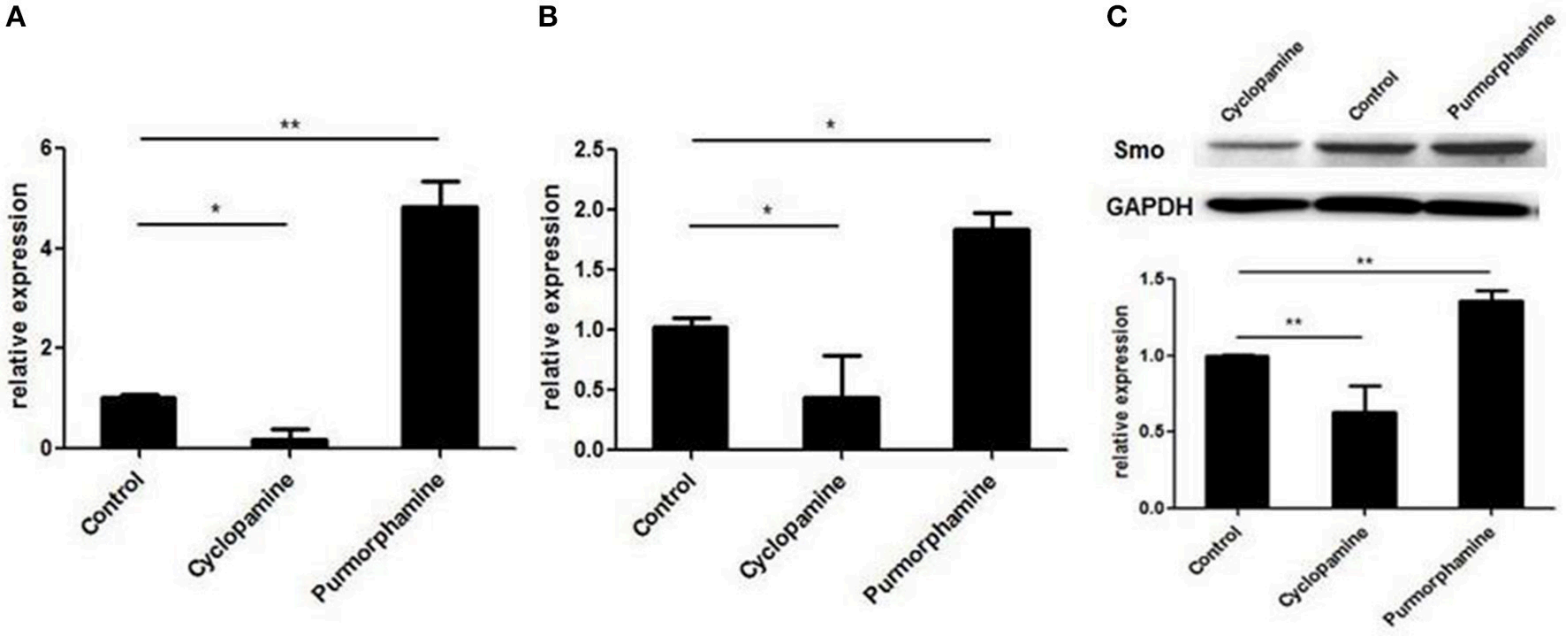
Purmorphamine and Cyclopamine regulate the activation of SHH signaling in RA-FLSs. FLSs were treated with Purmorphamine (1 μM) or Cyclopamine (10 μM). After 48 h of incubation, real-time PCR was used to determine GLI1 **(A)** and SMO mRNA expression **(B)**. Relative quantification of gene expression was performed by the 2^−ΔΔCt^ method. The expression of SMO protein was examined by western blot analysis and normalized to expression of GAPDH **(C)**. The results represent the mean ± SD based on ≥3 replicates.^*^P < 0.05 vs. control group. ^**^P < 0.01 vs. control group.

To investigate the effect of small molecules on SHH signaling of RA-FLSs, the expression of GLI1 mRNA and SMO mRNA were detected by the real-time PCR, and the expression of SMO protein was detected utilizing Western Blot. As shown in Figure 2, the expression of GLI1 mRNA, SMO mRNA, and SMO protein was up-regulated after treatment with the SHH agonist, Purmorphamine, and expression was decreased after incubation with SHH antagonist, Cyclopamine in RA-FLSs compared to the control group (all *P* < 0.05). The results suggest that the SHH agonist Purmorphamine and inhibitor Cyclopamine were fully functional and could independently activate or inhibit the SHH signaling pathway in RA-FLSs.

### The SHH Signaling Pathway Activates MAPK/ERK Pathway *in vitro*

**Figure 3:**
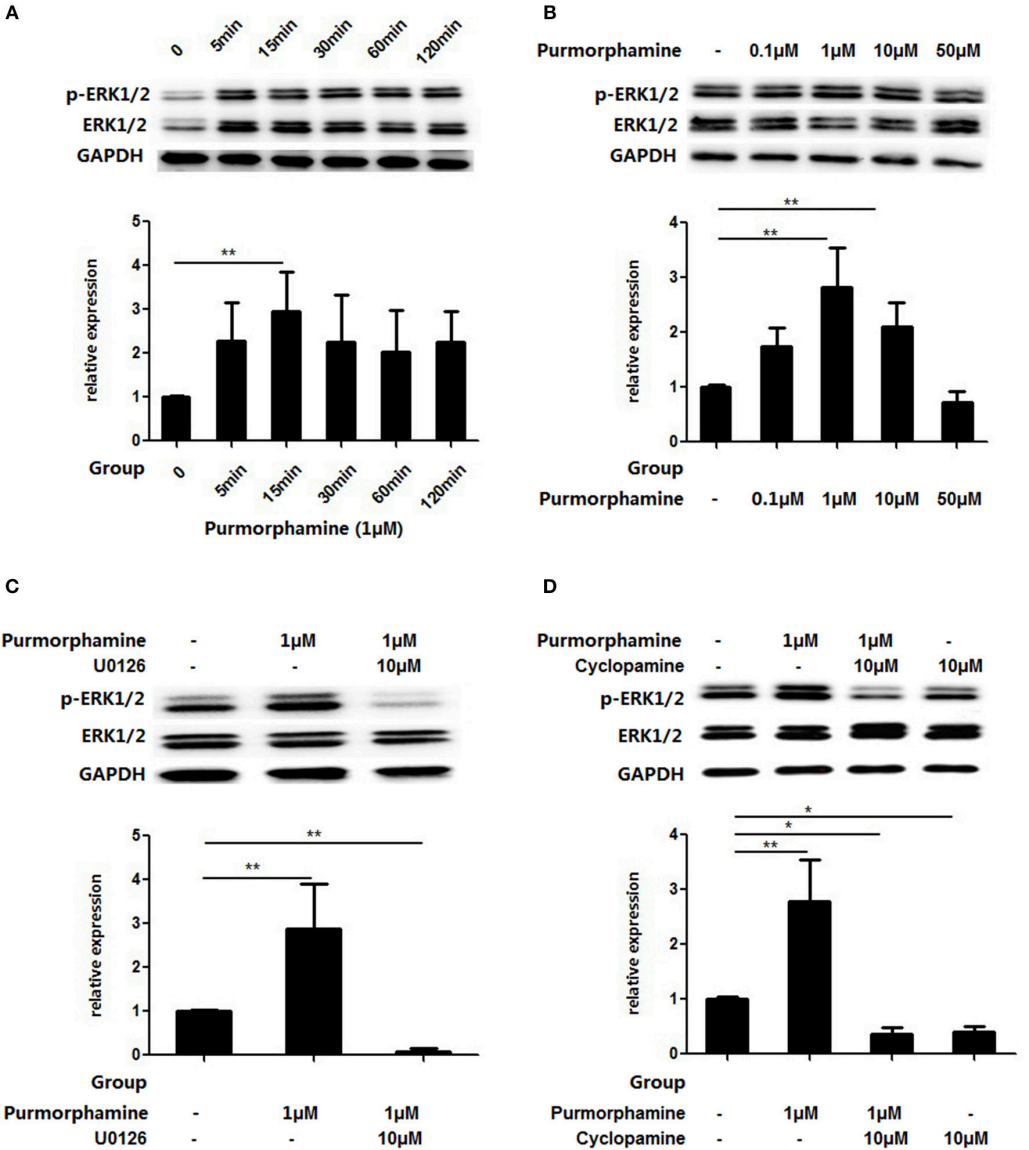
Purmorphamine and Cyclopamine regulate MAPK/ERK phosphorylation in RA-FLSs. FLSs were stimulated with 1 μM Purmorphamine for the indicated time **(A)** and various concentrations of Purmorphamine for 15 min **(B)**. The Purmorphamine-induced increases in MAPK/ERK phosphorylation were abolished in the presence of U0126-EtOH (10 μM) for 15 min **(C)**. Addition of Cyclopamine (10 μM) for 15 min abolishes the MAPK/ERK phosphorylation **(D)**. Expression of ERK1/2 was detected by western blot analysis and the levels of p-ERK1/2 were normalized to expression of total ERK1/2. The results represent the mean ± SD median values or interquartile ranges (IQR) based on ≥3 replicates. ^*^P < 0.05 vs. control group. ^**^P < 0.01 vs. control group.

Based on the above findings, we observed the influence of SHH signaling on the MAPK/ERK pathways in RA-FLSs. RA-FLSs were treated with Purmorphamine (1 μmol/ml) and the expression of p-ERK1/2 and total ERK1/2 was detected after different time points (5, 15, 30, 60, and 120 min). As shown in Figure 3A, compared with the control group, the expression of p-ERK1/2 in the total ERK1/2 protein was significantly increased at 15 min (*P* < 0.01). Then, RA-FLSs were treated with different concentrations of Purmorphamine (0.1, 1, 10, and 50 μM). After 15 min, the expression of p-ERK1/2 and total ERK1/2 was detected. Figure 3B showed that the proportion of p-ERK1/2 in total ERK1/2 was increased compared with the control group when RA-FLS were treated with 1 and 10 μM Purmorphamine (all *P* < 0.01). Furthermore, compared with the control group, Cyclopamine inhibited the phosphorylation of ERK1/2 (*P* < 0.05, Figure 3D), and the medians (interquartile range) of the proportion of p-ERK1/2 in total ERK1/2 is 0.42(0.29–0.48). SHH induced levels of p-ERK1/2 was inhibited by the MEK-specific inhibitor U0126-EtOH in the presence of Purmorphamine (*P* < 0.01, Figure 3C) compared with the control group, and the medians (interquartile range) of the proportion of p-ERK1/2 in total ERK1/2 is 0.06(0.02–0.13).

### SHH Signaling Promotes Proliferation of RA-FLS *via* the MAPK/ERK Pathway

**Figure 4:**
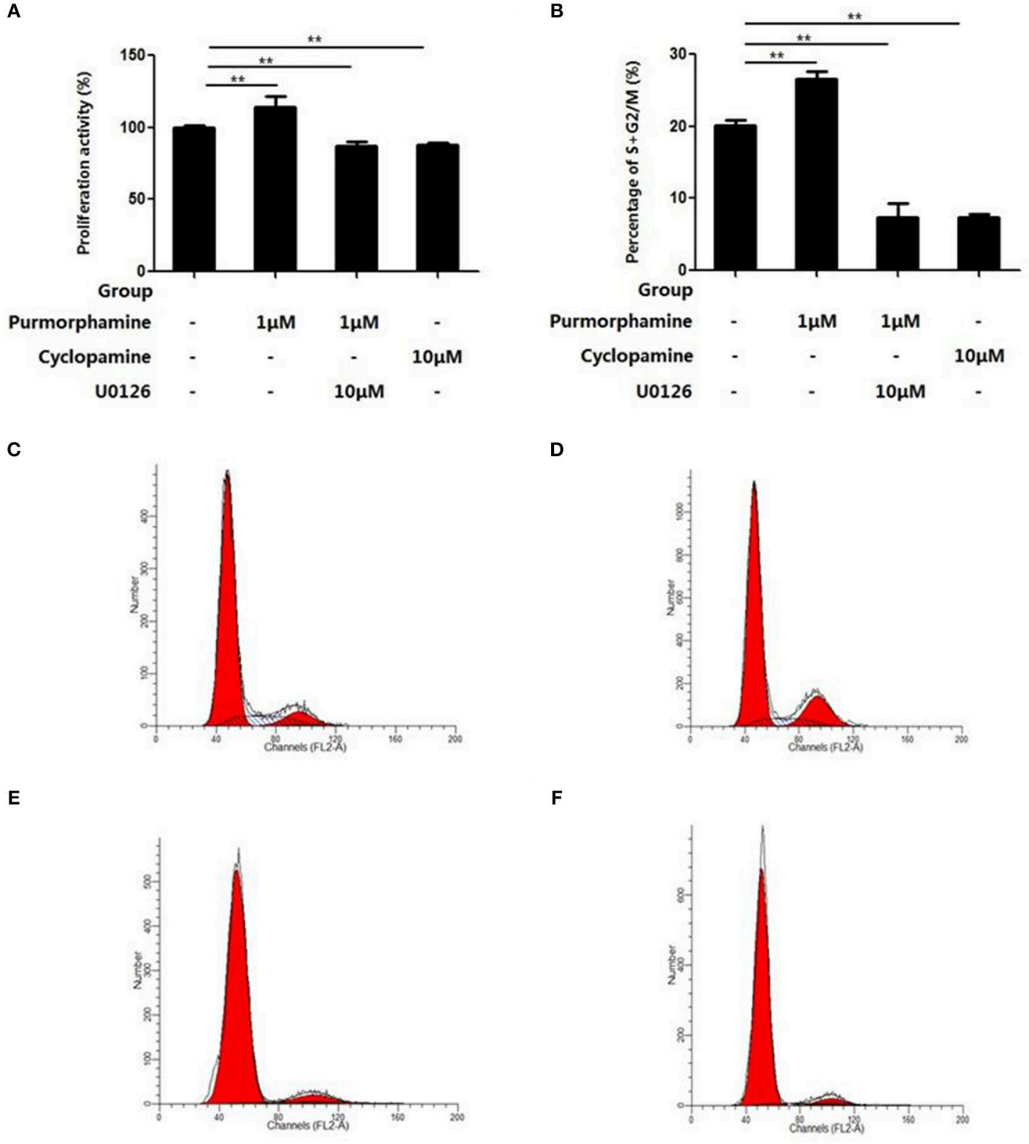
U0126-EtOH inhibit cell viability and regulate cell cycle distribution of RA-FLSs induced by SHH signaling. Cells were treated with vehicle (DMSO in DMEM supplemented with 10% FBS), Purmorphamine (1 μM), Cyclopamine (10 μM), or co-treated with U0126-EtOH and Purmorphamine cultured for another 48 h, respectively. Cell counting kit-8 (CCK-8) assay was performed to examine the cell viability **(A)**, and flow cytometry was used to analyze cells distribution **(B–F)**. The results represent the mean ± SD based on ≥3 replicates. ^**^P < 0.01 vs. control group.

The effects of U0126-EtOH, Cyclopamine, and Purmorphamine on cell viability was assessed by CCK-8 assays and flow cytometry was used to detect the cell cycle distribution. After the RA-FLSs were treated with Purmorphmine (1 μM) for 48 h, the cell viability was 114 ± 4%, and the percentage of G2/M+S phase cells was 26.57 ± 1.04%, higher than those of the control group 100 ± 0% (*P* < 0.01, Figure 4A) and 20.18 ± 0.68% (*P* < 0.01, Figure 4B), respectively. After RA-FLSs were treated with Cyclopamine (10 μM) for 48 h, and the viability of RA-FLSs was (88 ± 1)% (*P* < 0.01), the percentage of G2/M+S phase cells was (7.44 ± 0.44)% (*P* < 0.01). The effect of Purmorphamine (1 μM) on cell viability and cell cycle distribution was abolished in the presence of U0126-EtOH (10 μM), the cells viability of 87 ± 3% (*P* < 0.01) and the percentage ofG2/M+S phase cells was 7.31 ± 2.01% (*P* < 0.01) (Figure 4).

**Figure 5:**
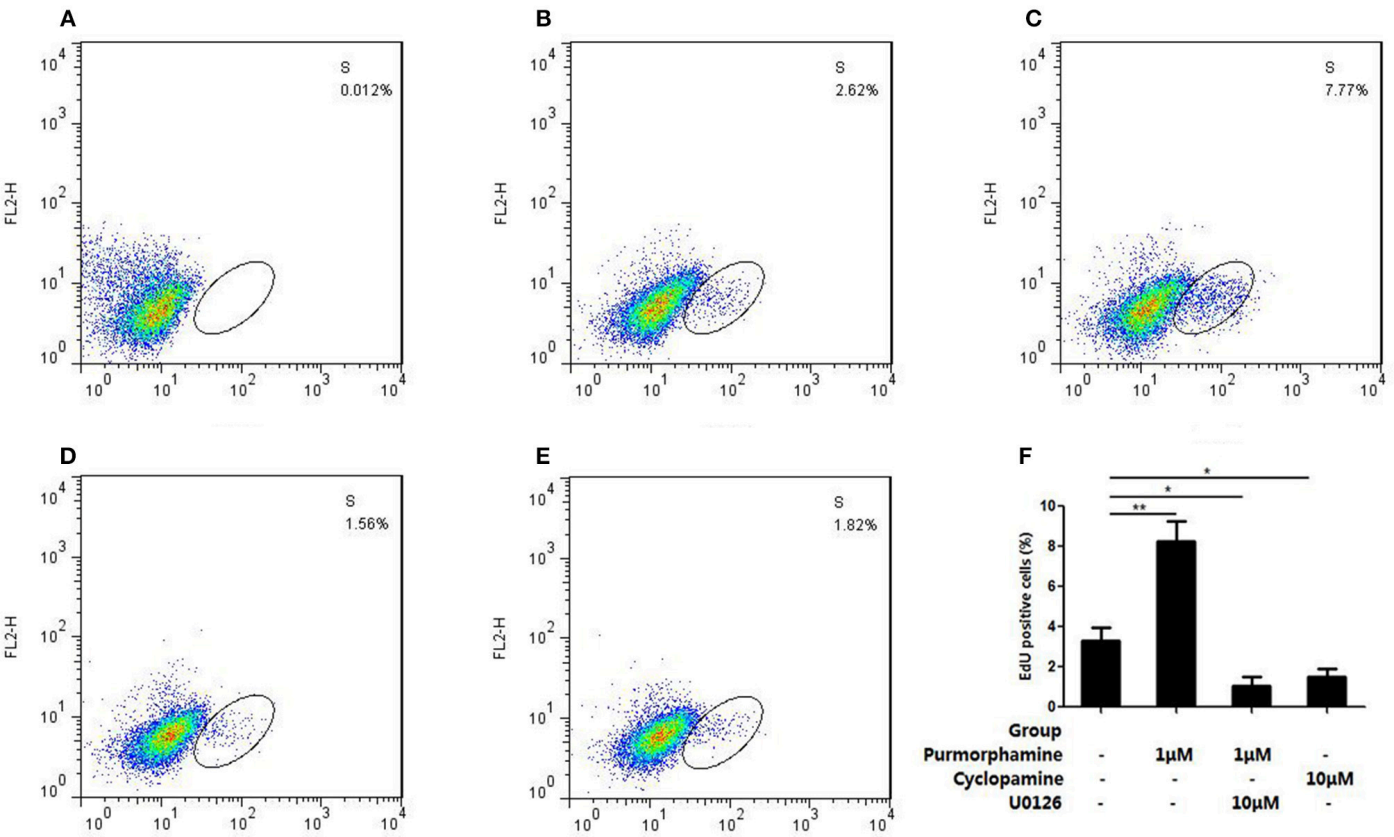
U0126-EtOH decrease the proliferating cells induced by SHH signaling. **(A)** Cells of the blank control group. **(B)** Cells were treated with vehicle (DMSO in DMEM supplemented with 10% FBS). **(C)** Cells were treated with Purmorphamine (1 μM). **(D)** Cells were co-treated with U0126-EtOH (10 μM) and Purmorphamine(1 μM). **(E)** Cells were treated with Cyclopamine (10 μM). The proliferating cells were detected by the flow cytometry and the ratio of EdU positive cells were calculated **(F)**. The results represent the mean ± SD based on ≥3 replicates. ^*^P < 0.05 vs. control group. ^**^P < 0.01 vs. control group.

In addition, flow cytometry analysis showed that incubation with Purmorphmine (1 μM) for 48 h resulted in a significant increase proliferating cells, and the ratio of proliferating cells is 8.24 ± 1.04% compared to controls 3.29 ± 0.69% (*P* < 0.01, Figures 5B,C). The ratio of proliferating cells were significantly decreased to 1.64 ± 0.45% (*P* < 0.05, Figure 5E) in the presence of Cyclopamine (10 μM). The effect of Purmorphamine (1 μM) on cell proliferation was abolished in the presence of U0126-EtOH (10 μM), with the ratio of proliferating was (1.07 ± 0.44)% (*P* < 0.05, Figure 5D). These results demonstrate that SHH signaling promotes proliferation of RA-FLSs *via* the MAPK/ERK pathway.

### SHH Signaling Promotes Migration of RA-FLS *via* the MAPK/ERK Pathway

**Figure 6:**
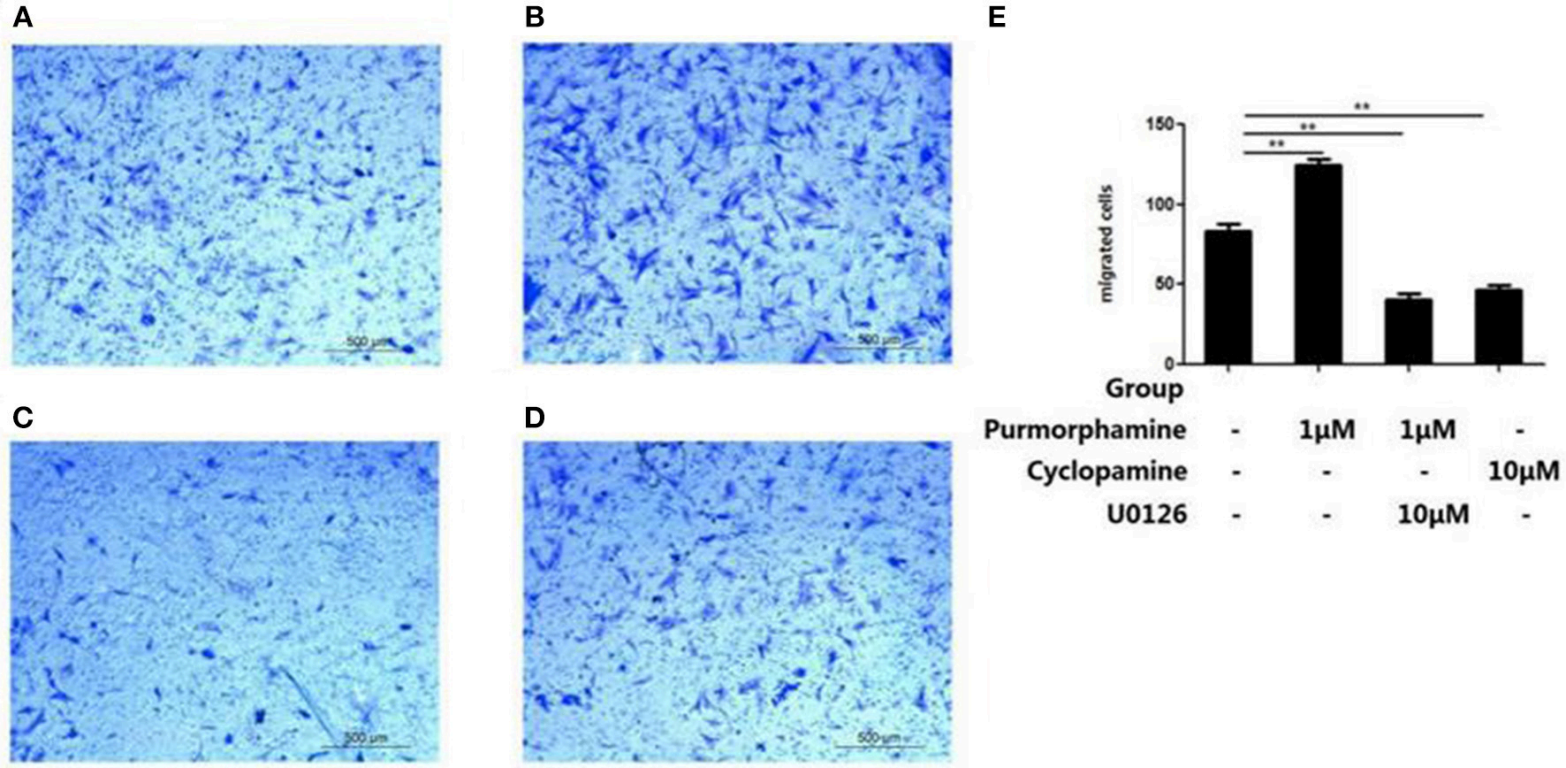
U0126-EtOH decrease the numbers of migration RA-FLSs induced by SHH signaling. Representative images of RA-FLSs migration after being treated with vehicle **(A)**, Purmorphamine (1 μM) **(B)**, co-treated with U0126-EtOH (10 μM) and Purmorphamine (1 μM) **(C)**, or Cyclopamine (10 μM) **(D)** were displayed (100 magnifications). Cell migration was evaluated using Transwell assays and the numbers of migrated cells were calculated (× 10^4^ cells/ml) **(E)**. The results represent the mean ± SD based on ≥3 replicates. ^**^P < 0.01 vs. control group.

To investigate the role of MAPK/ERK pathway in the SHH signaling-induced migration of RA-FLSs, Transwell migration assays were used to detect the effects of Purmorphamine, Cyclopamine and U0126-EtOH on cell migration. As shown in Figure 6E, incubation with Purmorphmine (1 μM) for 48 h significantly increased the number of migrated cells, with the number of migrated cells 124.67 × 10^4^ ± 3.51 × 10^4^, compared to that of the control group (83 × 10^4^ ± 4.58 × 10^4^, *P* < 0.01, Figures 6A,B). Incubation with Cyclopamine (10 μM) for 48 h significantly decreased the numbers of migrated cells to 46.33 × 10^4^ ± 3.21 × 10^4^ (*P* < 0.01, Figure 6D). We also observed that the effects of Purmorphamine on the migration of RA-FLSs was abolished in the presence of U0126-EtOH (10 μM), with the numbers of migrated cells 40.67 × 10^4^ ± 4.04 × 10^4^ (*P* < 0.01, Figure 6C). The numbers of migrated cells were not statistically different between Cyclopamine group and the co-treated (Purmorphamine and U0126-EtOH) group (*P* > 0.05).

## Discussion

RA-FLSs display a hyperplastic, aggressive and invasive phenotype, and are involved in the formation of pannus angiogenesis, cartilage degradation, and bone erosion (5). In our previous studies, we found that SHH signaling pathway plays a role in promoting RA-FLSs proliferation and migration (13, 14). However, the specific mechanism of SHH signaling pathway in the regulation of RA-FLSs proliferation and migration is still unclear. In this study, we present evidence that SHH signaling has effects on activating of MAPK/ERK signaling pathway. In addition, the SHH signaling pathway mediates the proliferation and migration of RA-FLSs *via* MAPK/ERK signaling pathway.

The SHH signaling pathway plays a vital role in early vertebrate development and in tumorigenesis. Aberrant activation of SHH signaling pathway is closely related to the occurrence and development of monoclonal proliferation in malignancy (19). In our previous studies, we found that SHH signaling pathway contribute to mediate the tumor-like behavior of FLSs depending on SMO (13). As a membrane protein ligand of SHH signaling pathway, SMO activation can positively initiates downstream signaling that results in the binding of GLI transcription factors to DNA and subsequently activates gene expressions (20). Cyclopamine interacts directly with the seven transmembrane helix structure of SMO, resulting in SMO conformation changes, inhibiting SHH signaling pathway (21). In the present studies, we used small molecules targets on SMO, agonist Purmorphamine and antagonist Cyclopamine. And we found that in RA-FLS the expression of SMO protein, SMO mRNA and GLI1 mRNA were up-regulated by Purmorphamine, and decreased under Cyclopamine treatment. The data suggests that the small molecules we used simulate the states of activation and inactivation of SHH signaling and may be useful to further investigate the mechanism of SHH signaling involved in proliferation and migration of RA-FLSs.

ERK1 and ERK2 are relevant protein-serine/threonine kinases that participate in the MAPK/ERK signal transduction cascade. Phosphorylation of two residues convert inactive ERK1/2 to the active form, which are the key molecules to transduce the signal from cell surface membrane to the nucleus (22). Aberrant activation of SHH pathway combined with functional mutations of K-RAS then stimulates the Raf/MEK/ERK cascade (23, 24). SHH signaling can regulate the expression level of p-ERK1/2 protein to activate the MAPK/ERK signaling pathway (25). In the present study, we investigated the relationship between MAPK/ERK signaling pathway and SHH signaling pathway in RA-FLSs, and found that Purmorphamine could increase the expression of p-ERK1/2 protein in concentration-and time-dependent manners. Also, Cyclopamine decreased the level of p-ERK1/2. Furthermore, the effects of Purmorphamine on activation of ERK1/2 were abolished in the presence of U0126-EtOH. The results suggest that SHH signaling pathway can affect the expression of p-ERK1/2, and further affect the transduction of MAPK/ERK signaling pathway in RA-FLSs. The membrane protein receptor SMO, is a member of the G protein-coupled receptor family depending on the Gα_i_ subunit (26). In addition to SMO activation, MAPK/ERK signaling pathway can also be activated by G protein-coupled receptor (27). In this study, our data suggest that SHH signaling regulates the activation of MAPK/ERK signaling pathway in RA-FLSs and further study is needed to determine whether SMO plays a role as G protein-coupled receptor in affecting the transduction of MAPK/ERK signaling pathway.

MAPK/ERK signaling pathway is closely related to the pathological development of RA. Schett et al. (28) found that MAPK/ERK signaling pathway is activated in T lymphocytes, FLSs and macrophages of synovial tissue in RA. Thiel MJ et al. (29) showed that the inhibition of MAPK/ERK signaling pathway significantly improves the symptoms of arthritis in CIA rats. IL-6 (30) and TNF-α (31) promote the proliferation of RA-FLSs *via* MAPK/ERK signaling pathway. Moreover, MAPK/ERK signaling pathway is also involved in the migration of RA-FLSs (32). Studies found that angiopoietin 1 (33), neuropilin-1, and vascular endothelial growth factor (34) regulate the lesion of rheumatoid joint and the proliferation of RA-FLSs *via* the MAPK/ERK signaling pathway. On the other hand, SHH signaling pathway regulates the occurrence and development of tumors *via* MAPK/ERK signaling pathway, such as liver cancer (16), gastric cancer (35), and non-small cell lung cancer (36). However, the role of MAPK/ERK pathway in the regulation of SHH signaling on activation of RA-FLSs remains unclear. In this experiment, we found that Purmorphamine can adjust the distribution of cell cycle, increase the proliferating cells and promote the proliferation of RA-FLSs. Furthermore, Purmorphamine can also promote the migration of RA-FLSs. However, we observed that the above phenomenon can be abolished by U0126-EtOH, and the inhibitory effect of U0126-EtOH treatment is not significantly different *in vitro* from that of Cyclopamine. Collectively, the data reported in this study have demonstrated that a novel link connecting SHH signaling with MAPK/ERK pathway to regulate the activation of RA-FLSs.

## Conclusion

In this study, we elucidated the relationship between SHH signaling pathway and MAPK/ERK pathway in RA-FLSs, and advanced the understanding of the molecular mechanism of SHH signaling pathway which is involved in proliferation and migration of RA-FLSs *via* MAPK/ERK pathway. These findings may provide important insights into the pathogenesis of RA and introduce a potential therapeutic target to suppress the aggressive activation of RA-FLSs.

## Author Contributions

JH, SGZ, and YP designed the experiments. FL, XF, SLZ, and YC performed the experiments. FL, XF, and SLZ analyzed these data. FL, JH, and HH wrote the manuscript. SGZ and RJ edited the manuscript.

### Conflict of Interest Statement

The authors declare that the research was conducted in the absence of any commercial or financial relationships that could be construed as a potential conflict of interest.
